# Medical Items

**Published:** 1917-07

**Authors:** 


### MEDICAL ITEMS

### SPEAKING OF OPIATES.

The objections to the employment of opium and morphine are two-fold. Following their use there usually is depression or partial suppression of normal functions, and if the drug is given hypodermically or in such fashion that its character is easily recognized by the patient the formation of a habit comes into the realm of the possible. To a large degree PAPINE (Battle) obviates these disadvantages. In the first place PAPINE (Battle) is prepared with the greatest care and from the purest drugs. The name is not indicative to the lay mind of its purposes and thus the patient is protected from falling into a habit. All in all, PAPINE (Battle) is the opiate of first choice.

---

**THE CONVENIENT ASPIROL.**

Aspirols afford ready means of medication by inhalation. They are convenient, practical different, the novelty appeals. The medicament in aspirols is enclosed in thin glass containers wrapped in braided silk covered absorbent cotton. Slight pressure fractures the glass; the wrapping absorbs the liquid and it is ready for inhalation.

Aspirols are manufactured by Eli Lilly & Company and the line includes Aspirols Ammonia, containing five mils of ammonia, useful in asphyxiation, fainting and other emergences; Aspirols Ammonia Aromatic, convenient to carry and serviceable in the office and sick room; and Aspirols Amyl Nitrate, used in angina pectoris, asthma and some forms of migraine. The latter are supplied in two sizes containing three and five minims.

Iodine Tubes and Ampoules of Iodine, the former containing two drops of tincture of iodine in a capillary glass tube, for sterilizing the site of hypodermatic injection, and the latter for purposes where more iodine is desired for painting, are both useful products that should be a part of every physician's armamentarium. These also are manufactured by Eli Lilly & Company and supplied through the drug trade.

---

In Neuritis, is the Hot Water Bottle the Best Anodyne? Palliation, by means of externally applied heat, is just as popular to-day as it was in Hippocrates' time.

The hot bath and the hot-water bottle are wonderful comforters. But who can be continuously in the bath tub, or who can be forever carrying a hotwater bottle? And how all too soon does the most faithful hot-water bottle lose its ardor and its temperature.

There is no simple adjunct in this category more simple and more genuinely effective than application by the patient himself, if possible along the course of the affected nerve, with K-Y ANALGESIC (methyl-salicylate, camphor and menthol, combined in a non-greasy, water-soluble base.)

K-Y ANALGESIC has the obvious advantage over the hot-water bottle in that "it stays put" for a much greater period of time. Nor is there the possible danger of a hot-water bottle burn—a factor especially to be thought of where the neuritis patient is weak and infirm.

In Pruritus—Even in severe forms of genital, anal, diabetic, eczematous itching, K-Y Lubricating Jelly in a great majority of cases, will bring relief, or at least grateful alleviation.

To anoint the skin in these conditions, K-Y Lubricating Jelly is not only effective, but convenient and economical, since it can be used without staining or soiling the bed clothes or the patient's linen. If the part is washed before each application, the best results are obtained.

---

The efficacy of antityphoid vaccination as a prophylactic measure in controlling typhoid fever has been clearly demonstrated from army hospital statistics. We have reached that stage of preventive medicine where it is possible to recommend the use of the vaccine with confidence and authority as a matter of personal hygiene which will practically insure protection against typhoid fever.

Elsewhere in this issue the Eli Lilly & Company urges physicians to protect their patients by vaccination against typhoid and paratyphoid fevers. Summer and autumn are the seasons when protective measures are most important, since it is then that persons are more likely to have accidental or unusual exposure and the use of Lilly Typhoid Vaccine, or Typhoid Mixed Vaccine is recommended as the best method of preventing the disease and reducing the danger of epidemics. Further particulars concerning Lilly Biologicals may be had by addressing the principal office of the company at Indianapolis and a handy Vest Pocket Manual on Biological Therapy will be sent to our readers on request.

### AMERICA'S AMPOULE HEADQUARTERS.

"I am glad that you carry Lilly Ampoules," said a physician to a retail druggist recently. "I always feel confident that if results are to be had from a preparation, I'll be certain of them if the product bears a Lilly label. That's why I prefer Lilly Ampoules."

The use of the ampoule is growing rapidly. The accuracy of dosage, directness of medication and convenience which ampoules afford appeal to the practitioner.

Eli Lilly & Company is said the manufacture the most complete line of ampoules made in this country. This well-known house has built up a reputation on ampoules that has won for it the sobriquet "The Ampoule House."

Lilly Ampoules are supplied individually wrapped and labeled in boxes of one-half and one dozen, also in substantial cloth covered slide-cases containing an assortment of ampoules in popular demand. They are supplied through the drug trade.

